# Influenza mRNA vaccine with engineered panhandle-forming UTRs provides potent, dose-sparing protection against seasonal influenza viruses

**DOI:** 10.1038/s41541-026-01463-3

**Published:** 2026-04-27

**Authors:** Yifan Xu, Xuehua Wan, Mingzhuo Chen, Benchi Li, Qian Weng, Qin Wang, Zhiliang Hu, Yongxiang Yi, Junwei Li

**Affiliations:** 1 Department of Infectious Disease, The Second Hospital of Nanjing, Affiliated to Nanjing University of Chinese Medicine, Nanjing, China; 2 Medical Innovation Center for Infectious Disease of Jiangsu Province, Nanjing, China; 3 Division of Hepatobiliary and Transplantation Surgery, Department of General Surgery, Nanjing Drum Tower Hospital, The Affiliated Hospital of Nanjing University Medical School, Nanjing, China

**Keywords:** Biotechnology, Diseases, Immunology, Microbiology

## Abstract

Influenza viruses pose a persistent threat to global public health, causing widespread respiratory illness and significant morbidity. Vaccination remains the most effective strategy to reduce the burden of both seasonal and pandemic influenza. mRNA vaccines represent a promising alternative to conventional vaccine platforms due to their rapid development, flexibility, and high efficacy. Nonetheless, optimizing non-coding regulatory elements such as untranslated regions (UTRs) remains crucial for enhancing mRNA vaccine performance. In this study, we designed a novel UTR derived from the influenza A virus M segment, engineered to form optimal panhandle structures through selective base-pair enhancing mutations (M1 + 2), aiming to improve mRNA translation efficiency and immunogenicity. Using both reporter and HA antigen-encoding mRNAs, we demonstrated that the M1 + 2 UTR significantly enhanced protein expression in vitro and in vivo compared to unmodified UTRs and the canonical α-globin UTR control. In a murine model, low-dose (0.1 μg) vaccination with HA mRNA-lipid nanoparticles (LNPs) incorporating the M1 + 2 UTR elicited robust innate, humoral, and T cell-mediated immune responses, and conferred complete protection against lethal challenge with seasonal influenza strains, including H1N1, H3N2, and IBV. Our findings underscore the potential of rational UTR design in developing more efficacious and dose-sparing mRNA vaccines.

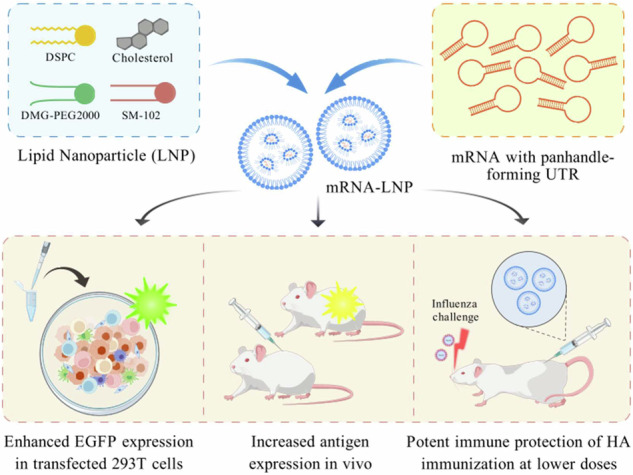

## Introduction

Despite the availability of licensed influenza vaccines, including inactivated (IIVs), live-attenuated (LAIVs) and recombinant influenza vaccines (RIVs)^1,2^, the continuous antigenic drift and shift of influenza viruses necessitate next-generation platforms with improved efficacy and broader protection^3^. In recent years, diverse influenza vaccine technologies have emerged, such as subunit vaccines, virus-like particle (VLP) vaccines, vector-based vaccines, nanoparticle vaccines, and nucleic acid vaccines^4–6^. Each of these approaches offers distinct advantages, such as broader cross-protective immunity^7,8^, enhanced immunogenicity^9,10^, or improved safety profiles^11^. However, limitations also exist including manufacturing complexity^12^, anti-vector immunity^13^, or potential risks of host genome integration^14^.

mRNA vaccines have emerged as a transformative platform for the prevention of infectious diseases including influenza, offering advantages such as rapid development, scalability, and the ability to induce potent humoral and cellular immune responses^15,16^. However, their efficacy and safety are influenced by several structural and immunological factors, including mRNA stability, translation efficiency, and inherent immunostimulatory properties^17^. A key strategy to enhance mRNA vaccine performance involves engineering the untranslated regions (UTRs), which play critical roles in regulating mRNA stability, subcellular localization, and translational activity^18–20^. Most current efforts have focused on human-derived UTR sequences to improve expression, yet viral UTRs which have evolved to support high-level protein synthesis and evade host immune responses represent a promising but underexplored alternative.

Of particular interest are UTRs from influenza viruses, which possess structured RNA elements that facilitate viral replication and translation^21,22^. The genomic RNA of influenza A virus is known to form panhandle structures through base-pairing between partially complementary 5’ and 3’ termini. These structures are essential for viral RNA transcription and replication, and have also been suggested to modulate translational efficiency and innate immune sensing^23^.

The panhandle structure is a conserved secondary structure formed by the reverse complementarity between the 5’ and 3’ terminal UTRs of viral genomic RNA during transcription^24^. It is widely present in many virus families, including *Orthomyxoviridae*, *Bunyaviridae*, *Reoviridae*, and *Arenaviridae*^25^. In influenza A virus, the panhandle structure has been directly visualized via microscopy and its secondary structure has been experimentally determined^26^. Beyond its well-characterized roles in viral RNA replication, transcription, packaging, and release^27^, emerging evidence suggested that panhandle structures can enhance the stability of circularized mRNA during translation initiation^25^. Furthermore, UTRs derived from viral genomes have been shown to stabilize mRNA and facilitate efficient translation of encoded proteins^21,28^. These features make panhandle-forming UTRs attractive candidates for improving mRNA vaccine design, as they may enhance antigen expression while fine-tuning immunostimulatory effects.

A significant challenge in mRNA vaccine development lies in balancing robust antigen production with appropriate innate immune activation. While excessive innate stimulation can lead to inflammation and adverse effects^17^, some immune sensing is necessary to initiate adaptive immunity. Current approaches to optimize mRNA vaccines include nucleoside modification such as pseudouridine incorporation to reduce innate immune activation and increase translation^29^, and improvements in lipid nanoparticle (LNP) formulations to enhance delivery and reduce reactogenicity^30,31^. Nevertheless, the deliberate incorporation of structured UTRs to simultaneously elevate translation and modulate immunogenicity remains poorly investigated.

In this study, we designed and screened UTRs derived from the influenza A virus M segment, which naturally form panhandle structures. Through rational mutagenesis to enhance base-pairing, we developed a novel UTR variant (M1 + 2) aimed at augmenting mRNA translation and tailoring innate immune activation. We evaluated its performance in mRNA-based influenza vaccines, demonstrating that the M1 + 2 UTR significantly enhances antigen expression in vitro and in vivo, improves immunogenicity at low doses, and confers broad protection against heterologous viral challenges. Our findings highlight the potential of structured viral UTRs in developing more efficacious and dose-sparing mRNA vaccines.

## Results

### Screening of UTRs and characterization of mRNA-LNPs

**Fig. 1:**
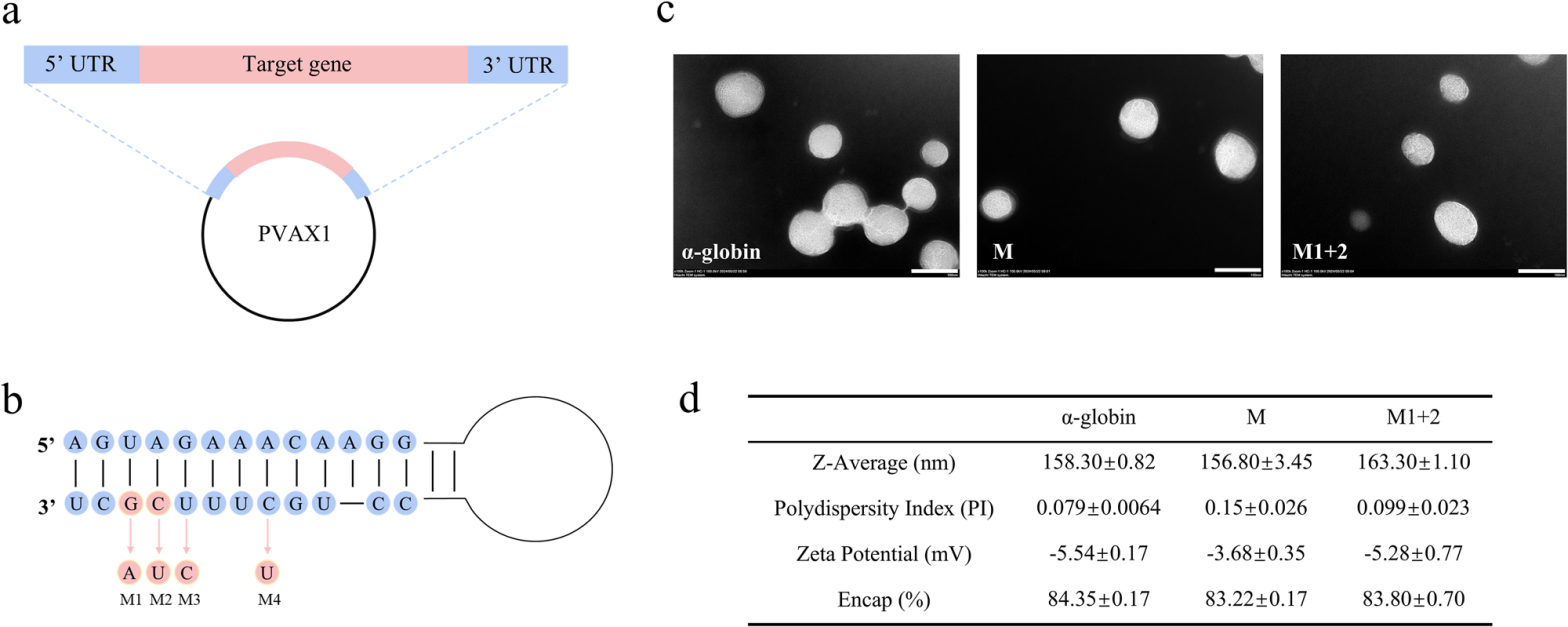
Schematic diagram of UTR variants and physicochemical characterization of mRNA-LNPs. **a** Schematic of the plasmid construct used for in vitro transcription of mRNA. **b** Schematic of the influenza M segment-derived UTR secondary structure, indicating sites of mutagenesis (M1, M2, M3, and M4) introduced to enhance base-pairing within the panhandle domain. **c** Representative negative-stain transmission electron microscopy (TEM) image of mRNA-LNPs showing spherical morphology (scale bar: 100 nm). **d** Hydrodynamic diameter, polydispersity index (PDI), zeta potential, and encapsulation efficiency of mRNA-LNPs as determined by dynamic light scattering (DLS) and RiboGreen assay, respectively. Data are presented as mean ± SD (*n* = 3).

The UTRs of mRNA play a critical role in regulating stability, localization, and translational efficiency. To identify potent UTRs for enhancing mRNA vaccine expression, we used EGFP as a reporter to screen a series of UTR sequences derived from viral gene fragments of hantavirus, rotavirus, influenza A virus, and others, capable of forming panhandle structures (Fig. 1a, b). The UTR from the influenza A virus M gene segment demonstrated the highest efficiency in promoting GFP expression (Fig. S1). To further optimize its performance, we introduced a series of mutations into the 3’ UTR of the M segment to increase base-pairing (Fig. 1b). mRNA was synthesized via in vitro transcription (IVT) using a T7 promoter. LNPs were formulated with SM-102, DSPC, cholesterol, and DMG-PEG2000 at a molar ratio of 50:10:38.5:1.5 in ethanol. The mRNA and lipid mixture were combined using a microfluidic mixer at an ethanol-to-aqueous phase ratio of 1:3. The encapsulation efficiency of mRNA in LNPs, as determined by the RiboGreen assay, exceeded 80% (Fig. 1d). Negative-stain transmission electron microscopy (TEM) revealed that the mRNA-LNPs exhibited spherical morphology with a diameter of approximately 100 nm (Fig. 1c). Consistent with this, dynamic light scattering (DLS) measurements indicated a particle size of approximately 100 nm with a narrow polydispersity index (PDI) below 0.1 (Fig. 1d). Agarose gel electrophoresis revealed that mRNAs produced a single, clear band with no visible degradation or smearing (Fig. S2). The comparable band intensity and absence of lower molecular weight fragments confirmed that all mRNAs were full-length and intact prior to encapsulation. These results demonstrate the successful preparation of well-characterized mRNA-LNP formulations suitable for downstream evaluation.

### mRNA-LNPs Containing M1 + 2 UTR Exhibit Enhanced In Vitro Translational Efficiency

**Fig. 2:**
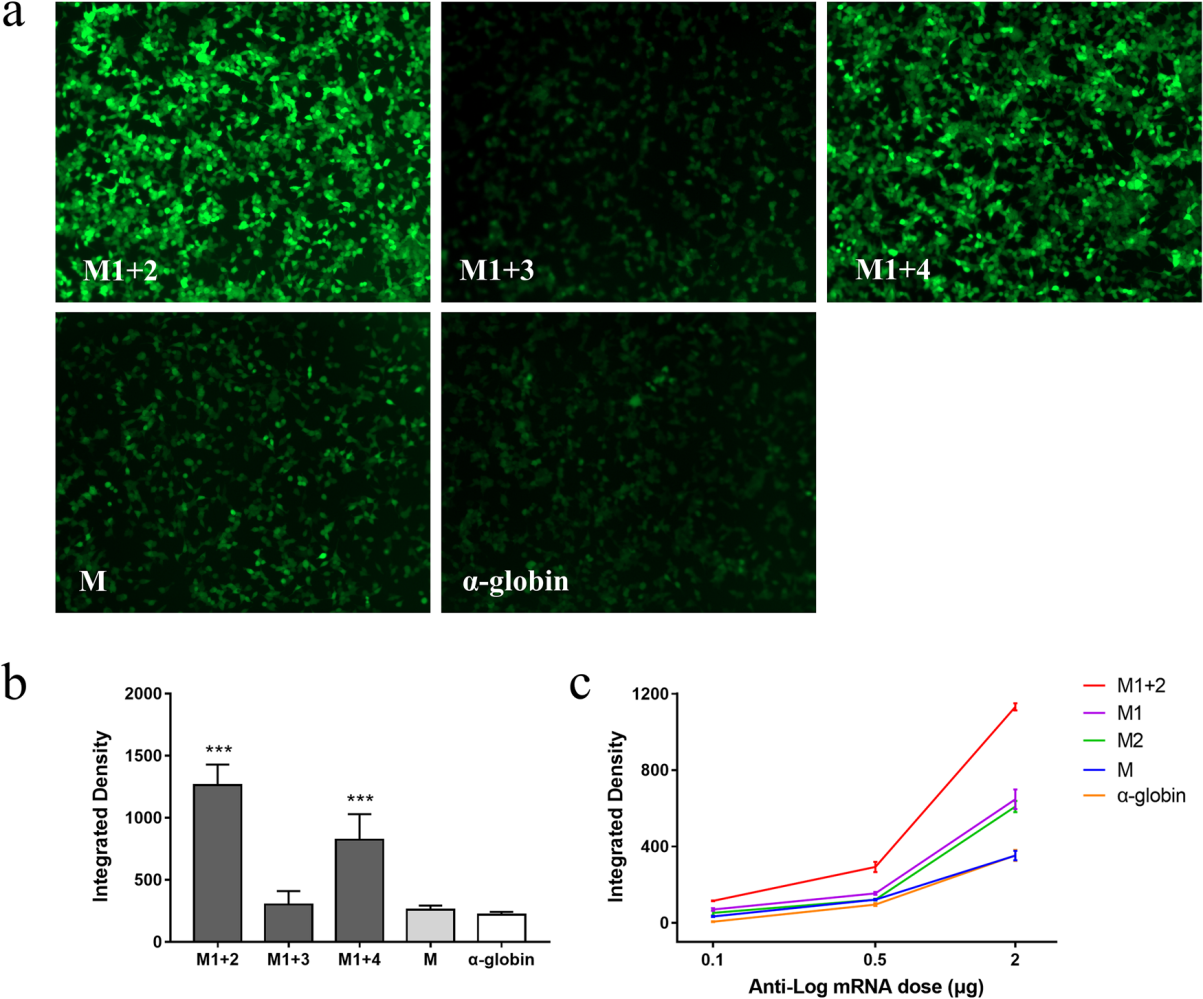
M1 + 2 UTR enhances in vitro translation efficiency of mRNA-LNPs. **a** Representative fluorescence microscopy images of HEK-293T cells taken at 24 h after transfection with 2 μg of EGFP-encoding mRNAs formulated in LNPs. The mRNAs contained different UTR variants: prototype M segment UTR (M), engineered variants (M1 + 2, M1 + 3, M1 + 4), and human α-globin UTR as a control. **b** Quantification of total EGFP fluorescence intensity from images in (**a**) expressed as integrated density per image field measured using ImageJ. Cells were transfected with 2 μg mRNA and imaged 24 h post-transfection. **c** Dose-dependent EGFP expression at 24 h after transfection with 2, 0.5, or 0.1 μg mRNA. For each dose, fluorescence images were captured by microscopy and total fluorescence intensity was quantified as integrated density per image field using ImageJ. Data are presented as mean ± SD (*n* = 3); *: *P* < 0.05, **: *P* < 0.01, ***: *P* < 0.001, compared with α-globin group.

In order to systematically evaluate the role of panhandle structure in mRNA translation and protein production, we introduced several mutations (M1, M2, M3, and M4) into the UTR of the influenza M gene segment (Fig. 1b), using the α-globin UTR as a control. Transfection of HEK293T cells with mRNA-LNPs containing these UTRs revealed that the M1 mutation (a G-to-A substitution) significantly enhanced EGFP expression (Fig. S3). Building on this, we systematically introduced additional mutations (M2, M3, M4) and found that the M1 + 2 combination yielded the most pronounced increase in EGFP fluorescence (Fig. 2a, b). This enhancement was consistent across three different transfection doses (2, 0.5, and 0.1 µg), with the M1 + 2 UTR consistently showing the strongest fluorescence, significantly outperforming the prototype M, single M1 or M2 mutations, and the linear α-globin UTR control (Fig. 2c) (see Fig. S4 for representative images and complete quantification). Computational calculation of UTR secondary structures revealed that the introduced mutations alter thermodynamic stability, although the predicted ΔG values did not strictly correlate with translational efficiency (Fig. S5), consistent with the notion that functional performance was decided by specific structural features rather than absolute stability. These findings demonstrated that the specific conformation of a panhandle structure formed by M1 + 2 UTR makes it the optimal variant, significantly outperforming all other tested UTRs in enhancing protein expression in vitro.

### mRNA-LNPs with M1 + 2 UTR enhance in vivo antigen expression

**Fig. 3:**
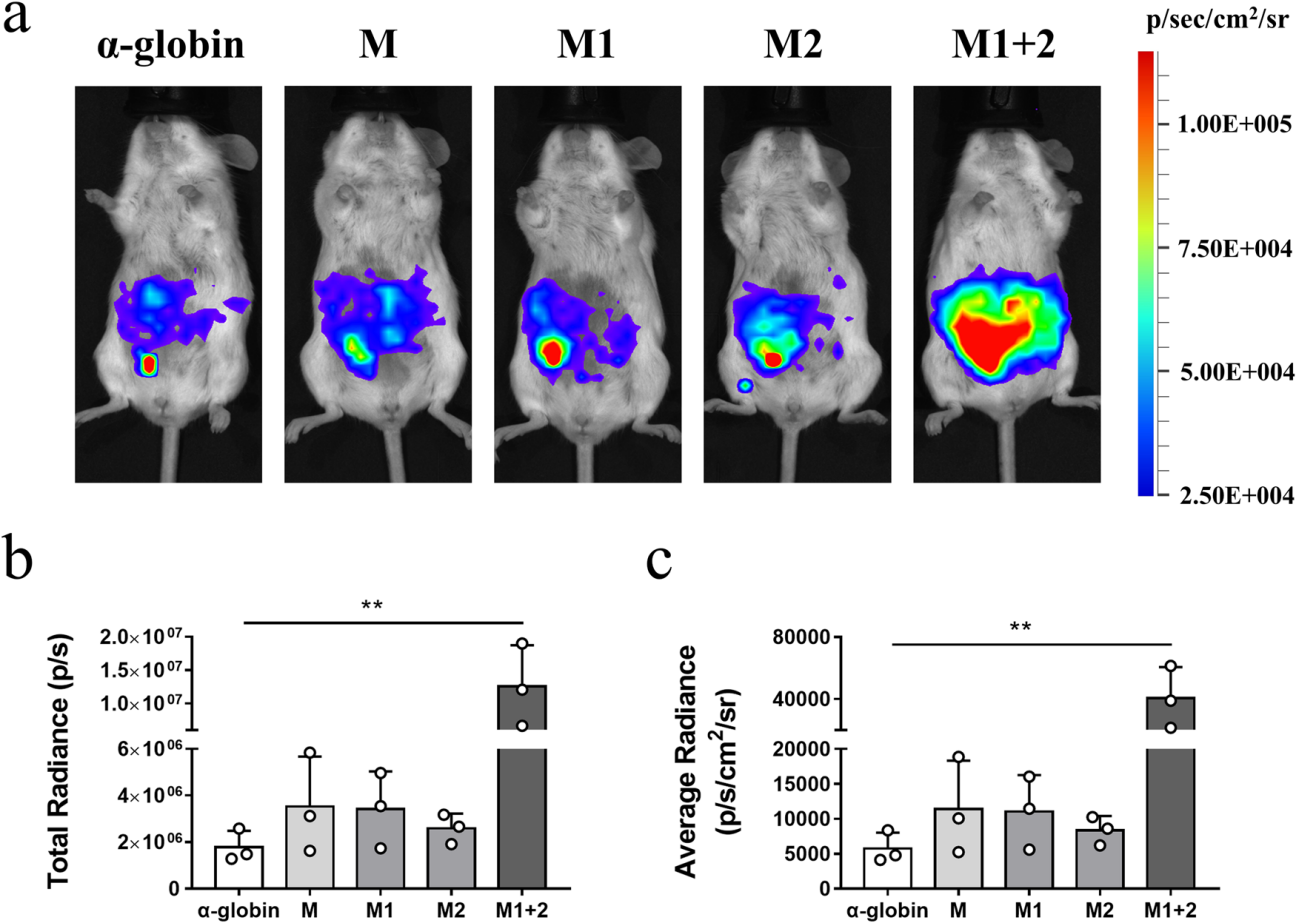
M1 + 2 UTR enhances in vivo luciferase expression. **a** Representative bioluminescence images of BALB/c mice taken at 48 h after intramuscular injection of Luc-mRNA-LNPs containing the indicated UTR variants (α-globin, M, M1, M2, and M1 + 2). Mice were administered luciferase substrate intraperitoneally 10 min prior to imaging. (b, c) Quantification of total flux (photons/s) (**b**) and average radiance (photons/s/cm²/sr) (**c**) within the region of interest. Data are presented as mean ± SD (*n* = 3). **: *P* < 0.01, compared to the α-globin control group.

To determine whether the improvements observed in vitro translate to an in vivo setting, we packaged luciferase mRNA bearing different UTRs into LNPs and administered them intramuscularly to mice. Bioluminescence imaging revealed that the signal was predominantly localized in the abdominal region. This distribution pattern is consistent with multiple independent reports demonstrating that intramuscularly administered mRNA-LNPs can undergo lymphatic drainage to regional lymph nodes, which are a primary component of the abdominal signal^32–36^. In full agreement with our findings, a recent study^32^ reported that at 2 days after intramuscular injection of luciferase-encoding mRNA-LNPs, the bioluminescence signal was predominantly detected in the abdominal region. The pronounced abdominal signal pattern virtually identical to our observations here. The signal we observed, therefore, represents robust protein expression at these sites. In our research, luciferase expression was significantly higher for all panhandle-forming UTRs compared to the linear α-globin control (Fig. 3a). Notably, the M1 + 2 UTR induced the strongest luminescence signal, significantly outperforming the α-globin UTR and closely matching the hierarchy observed in vitro (Fig. 3b, c). This confirms that the M1 + 2 UTR significantly enhances antigen expression not only in cultured cells but also in living animals.

### mRNA-LNPs with M1 + 2 UTR elicit potent antigen-specific antibody responses

**Fig. 4:**
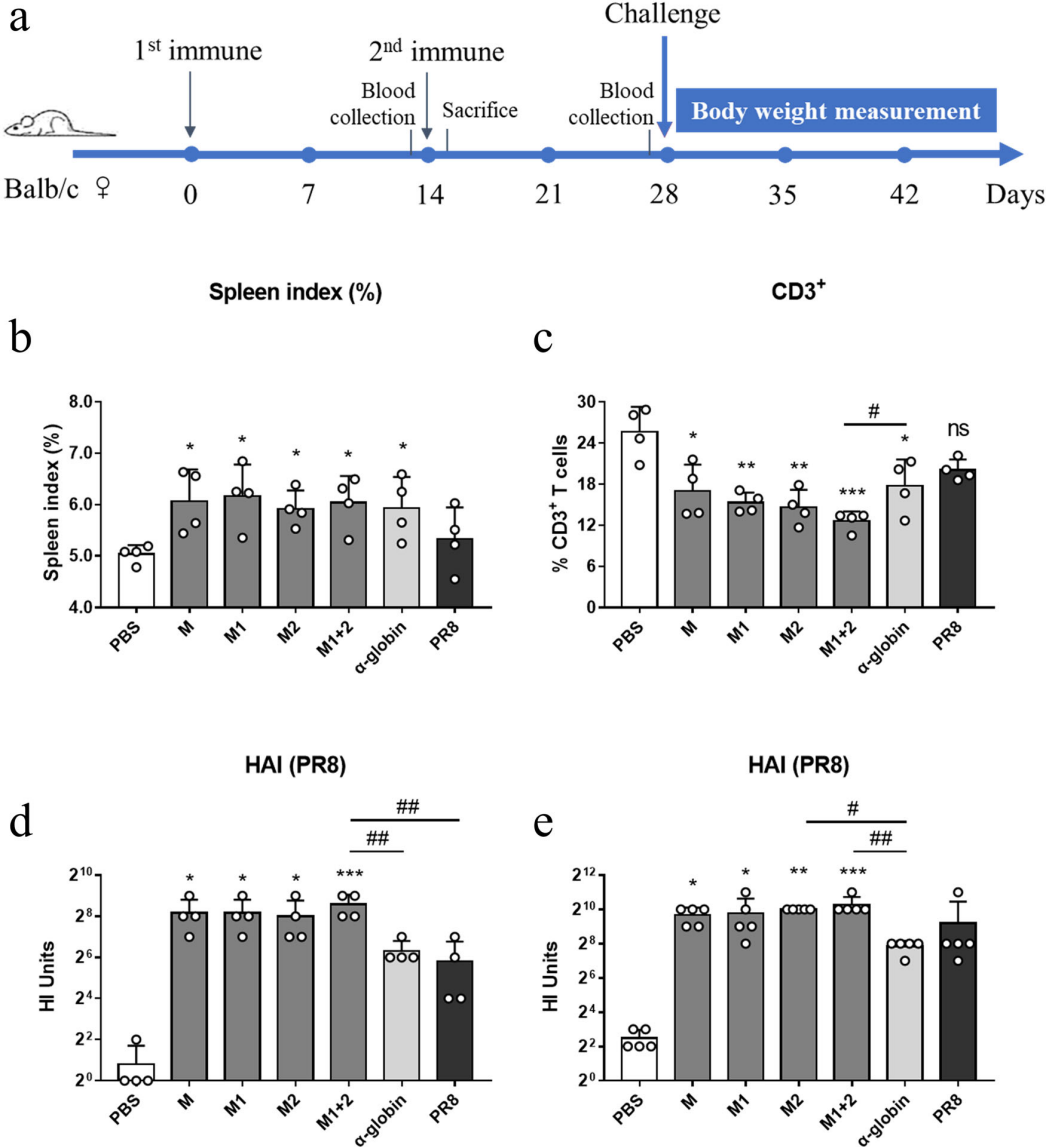
M1 + 2 UTR elicits enhanced humoral immune responses and robust adaptive immunity. **a** Schematic diagram of the immunization and sampling schedule. Female BALB/c mice were immunized intramuscularly with mRNA-LNPs on Day 0 and Day 14. Blood was collected at indicated time points. Mice were challenged on Day 28 and monitored for body weight changes thereafter. **b** Spleen index (spleen weight/body weight × 100%) measured at 24 h post-boost immunization. **c** Flow cytometric analysis of CD3⁺ T cell populations in splenocytes at 24 h post-boost. Hemagglutination inhibition (HI) antibody titers against the PR8 influenza virus measured at Day 13 (post-prime, **d**) and Day 27 (post-boost, **e**). Data are presented as mean ± SD (*n* = 4–5 per group); *: *P* < 0.05, **: *P* < 0.01, ***: *P* < 0.001, compared with PBS control group; #: *P* < 0.05, ##: *P* < 0.01, compared with α-globin UTR group.

Having established the enhanced expression profile of the M1 + 2 UTR, we evaluated its performance in a vaccine context. Six-to-eight-week-old BALB/c mice were immunized intramuscularly with H1N1 (PR8) HA mRNA-LNP vaccines according to the schedule in Fig. 4a. A prime-boost regimen (14-day interval) was used. At 24 h post-boost, the spleen index was significantly increased compared to the PBS group (Fig. 4b), indicating immune activation. The analysis of splenocytes at 24 h post-secondary immunization was specifically chosen to capture the early recall and activation of the immune response following antigen re-exposure, rather than to assess the peak of adaptive immunity. Flow cytometric analysis of splenic PBMCs revealed no significant differences in CD4^+^/CD8^+^ T cell subsets among groups (Figs. S6, S7). However, we observed a significant reduction in the proportion of CD3^+^ T cells in mice immunized with HA mRNA-LNPs or infected with PR8 virus, with the most pronounced decrease seen in the M1 + 2 group (Fig. 4c). Given that the majority of murine splenocytes are lymphocytes, and considering that the observed reduction in CD3^+^ T cell proportion inversely correlates with the superior antibody production in the M1 + 2 group (as shown in Fig. 4d, e), this shift in lymphocyte composition strongly suggests a concomitant expansion of non-T cell populations. Since B cells constitute the major lymphocyte population in the murine spleen^37^, our data imply that the M1 + 2 UTR most effectively promotes an environment conducive to B cell expansion and the subsequent enhanced antibody production.

Hemagglutination inhibition (HI) titers were tested to compare the levels of HA-specific antibodies in the serum of mice immunized with HA mRNA-LNP vaccine. HI assays revealed that mice immunized with panhandle-UTR vaccines developed higher HI titers than those infected with live PR8 virus (Fig. 4d, e). Importantly, the M1 + 2 construct consistently trended toward the highest HI titer. It indicates the M1 + 2 UTR construct induced a particularly strong humoral response. Notably, at this 2 μg dose, the HI titers induced by the M1 + 2 UTR construct, while high, were not significantly different from those achieved by other lead candidates. We hypothesized that this standard dose might be sufficient to elicit a maximal immune response, thereby masking the incremental advantage in translational efficiency offered by the M1 + 2 UTR. This ceiling effect will be further contextualized following the viral challenge outcomes (see Fig. S9) and investigated in a subsequent low-dose experiment.

### mRNA-LNP vaccine with M1 + 2 UTR potentiates cellular immune responses

**Fig. 5:**
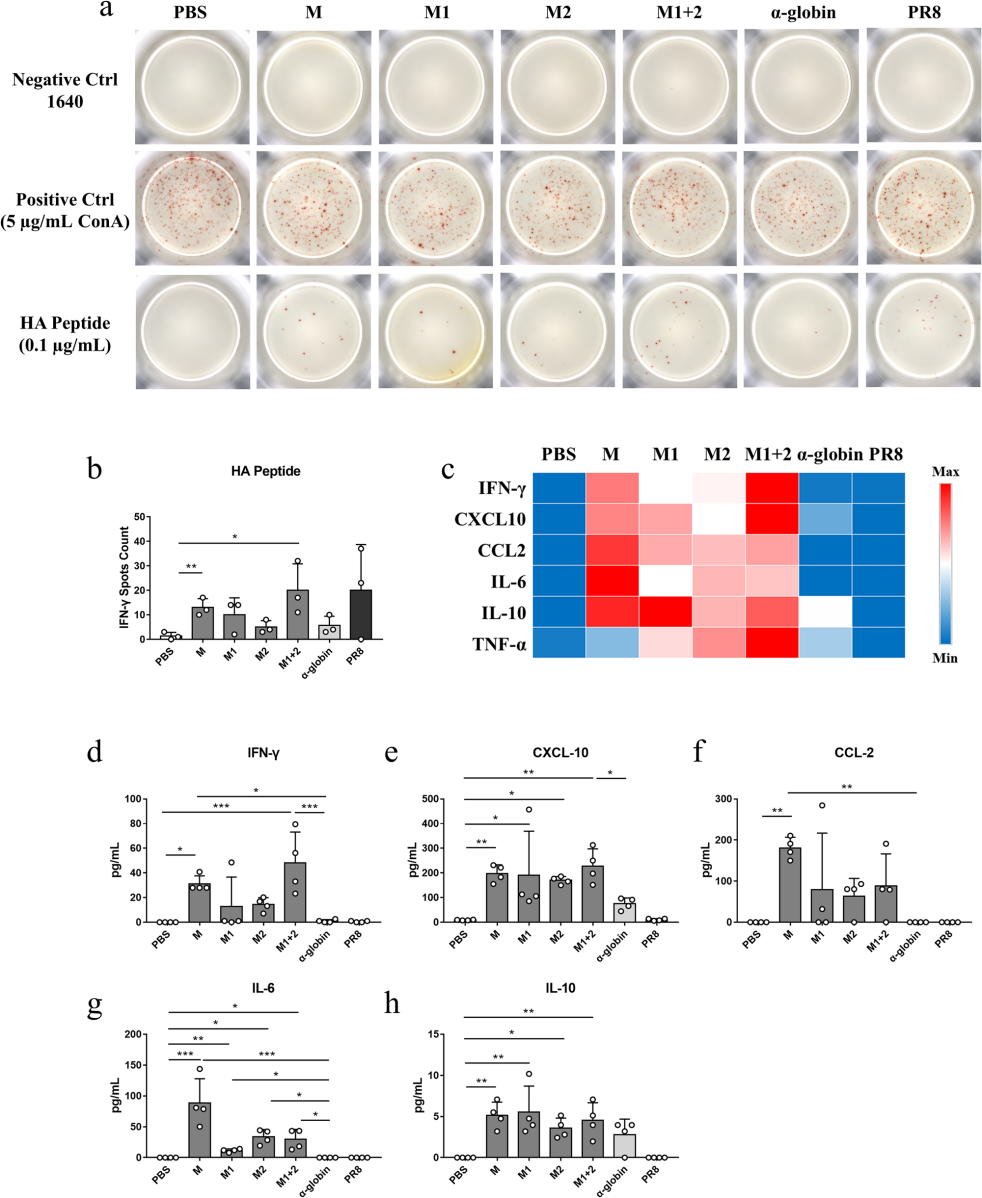
M1 + 2 UTR promotes cellular immune responses and cytokine production. **a** Representative ELISPOT images of IFN-γ⁺ splenocytes isolated from mice at 24 h post-boost immunization. Cells were stimulated with RPMI 1640 medium only (negative control), ConA (positive control), or HA peptide (specific activator). **b** Quantification of IFN-γ spot-forming cells after HA peptide stimulation (related to the ELISPOT images in **a**). **c** Heatmap showing serum levels of cytokines and chemokines measured by Luminex assay at 24 h post-boost. Data represent the mean value of most significantly altered analytes across groups. Quantitative analysis of IFN-γ (**d**) CXCL10 (**e**) CCL2 (**f**) IL-6 (**g**) and IL-10 (**h**) in serum from the indicated groups. Data are presented as mean ± SD (*n* = 3–4). *: *P* < 0.05, **: *P* < 0.01; ***: *P* < 0.001; specific pairwise comparisons are indicated in the figure.

We next sought to determine whether our vaccine platform could elicit a rapid antigen-specific T cell recall response shortly after booster immunization. Splenocytes were harvested 24 h post-boost and subjected to IFN-γ ELISPOT assay. The time point was chosen to capture early immune reactivation rather than the peak of T cell expansion. Critically, the antigen-specific spots detected in HA-peptide stimulated wells were absent in the mock-stimulated (1640 medium-only) background controls across all immunized groups except PBS-control group (Fig. 5a), confirming the specificity of the responses. As quantified in Fig. 5b, both the M and M1 + 2 vaccine groups induced a significant, albeit modest, increase in IFN-γ spot-forming units compared to control group, with the M1 + 2 construct proving to be the most effective. This result indicates that vaccination with M1 + 2 mRNA-LNPs primes a T cell pool that can be rapidly re-activated upon re-encounter with the antigen, which is a key feature of an effective vaccine.

In addition to T-cell responses, we profiled systemic cytokine levels using a Luminex assay. Mice immunized with mRNAs containing panhandle-structured UTRs (M, M1, M2, and M1 + 2) showed elevated concentrations of IFN-γ, CXCL-10, CCL-2, IL-6, IL-10, and TNF-α (Fig. 5c). Specifically, the M and M1 + 2 vaccine groups induced significantly higher cytokine production compared to the PBS group, and also surpassed levels observed in the α-globin UTR and PR8 infection groups (Fig. 5d–h). These findings suggest that UTRs capable of forming panhandle structures not only promote enhanced immunogenicity but also amplify the intrinsic adjuvant effect of mRNA by stimulating innate immune activation.

### HA mRNA-LNPs with M1 + 2 UTR confer dose-sparing protection against seasonal influenza challenge

To evaluate the protective efficacy of the HA mRNA-LNP vaccines, immunized mice were intranasally challenged with a lethal dose of influenza virus PR8 at 14 days after the boost immunization of 2 μg per mouse. Mice in the PBS control group exhibited severe influenza-like symptoms, including ruffled fur, significant weight loss, reduced appetite, and lethargy (Fig. S9a). All mice in the PBS group ultimately succumbed to infection, whereas all animals vaccinated with HA mRNA-LNP vaccines, regardless of UTR variant, survived the challenge without overt signs of disease (Fig. S9b), demonstrating that vaccination conferred robust protection.

Although the M1 + 2 UTR variant demonstrated superior efficacy in enhancing the translation of reporter genes (EGFP and Luc) in both in vitro and in vivo settings (Fig. 2 and 3), its advantage in conferring protective immunity following a standard 2 μg vaccine dose was less pronounced. All vaccinated groups, including those receiving mRNAs with other UTR variants, generated robust HI antibody titers (Fig. 4d, e) and were fully protected against lethal viral challenge, as evidenced by survival rates and minimal weight loss (Fig. S9). We reasoned that this standard dose might be supra-threshold, eliciting a maximal protective response that could mask more subtle advantages in translational efficiency. To investigate whether the enhanced translational capacity of the M1 + 2 UTR could provide a tangible benefit under more stringent conditions, we conducted a subsequent vaccination study utilizing a reduced mRNA dose regimen.

**Fig. 6:**
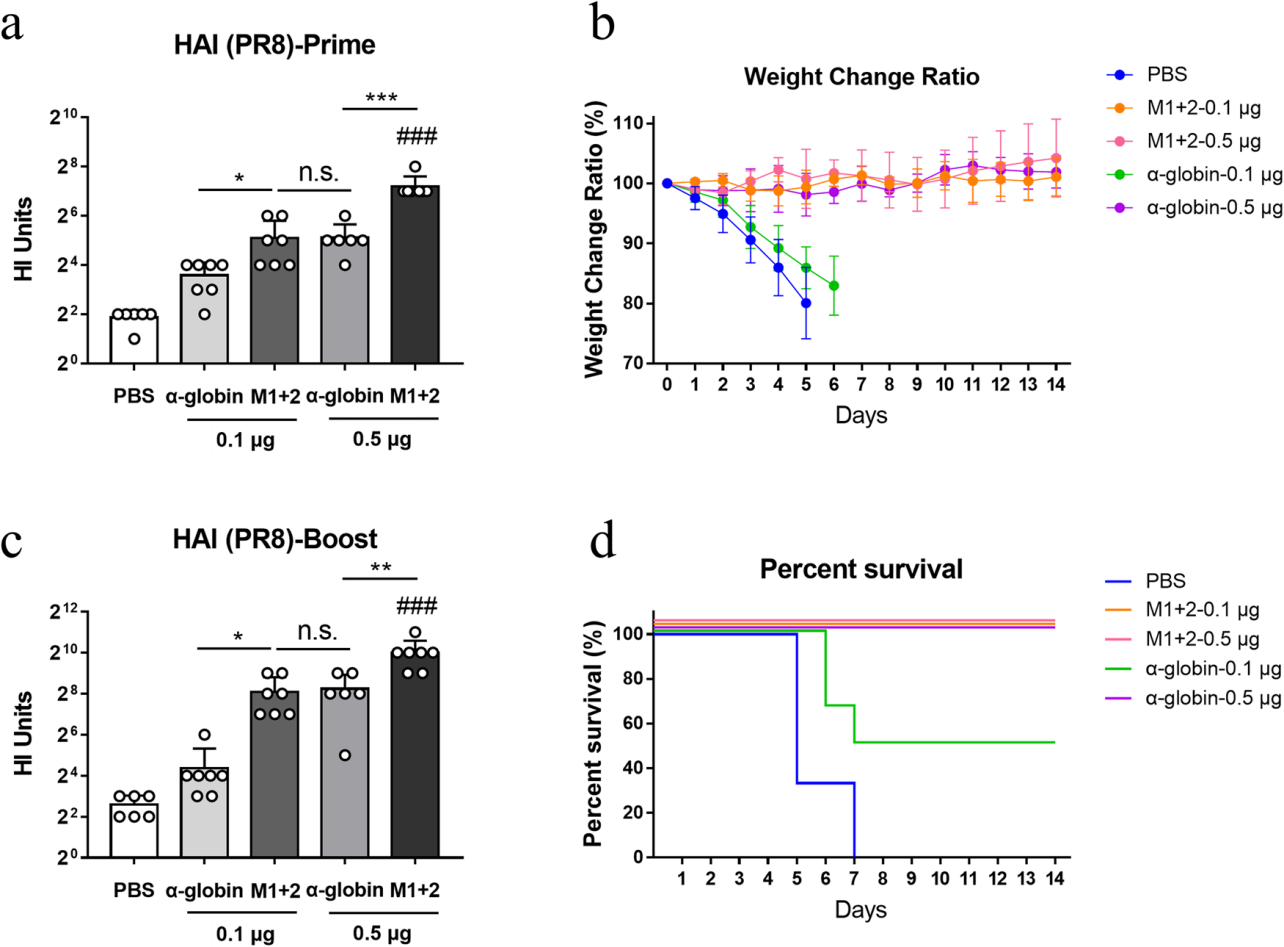
M1 + 2 UTR confers dose-sparing protection against viral challenge. HI antibody titers against the PR8 influenza virus measured at 14 days after prime (**a**) and boost (**c**) immunizations with 0.1 or 0.5 μg of mRNA-LNPs containing the indicated UTR variants. **b** Body weight changes of mice following challenge with PR8 (H1N1) virus. Mice were monitored daily for 14 days post-challenge; data are presented as mean percentage of initial body weight. **d** Survival rates of the same mice over the 14-day observation period. Data are presented as mean ± SD (*n* = 6–7). n.s.: not significant (*P* ≥ 0.05); ###: *P* < 0.001, compared with PBS; *: *P* < 0.05, **: *P* < 0.01; ***: *P* < 0.001, compared with α-globin.

Mice were immunized with two doses of 0.1 μg or 0.5 μg of HA mRNA-LNPs bearing either the M1 + 2 or α-globin UTR. Following challenge, animals that received 0.1 μg of the α-globin UTR vaccine exhibited marked weight loss and clinical signs of infection (Fig. 6b). In contrast, those immunized with 0.1 μg of M1 + 2 UTR vaccine elicited high HI titers after both prime and boost immunizations (Fig. 6a, c) and were completely protected from challenge, with no weight loss and 100% survival. The α-globin group at the same dose showed only 87.5% survival (Fig. 6d), indicating that the M1 + 2 UTR provides stronger protective immunity at low antigen doses.

**Fig. 7:**
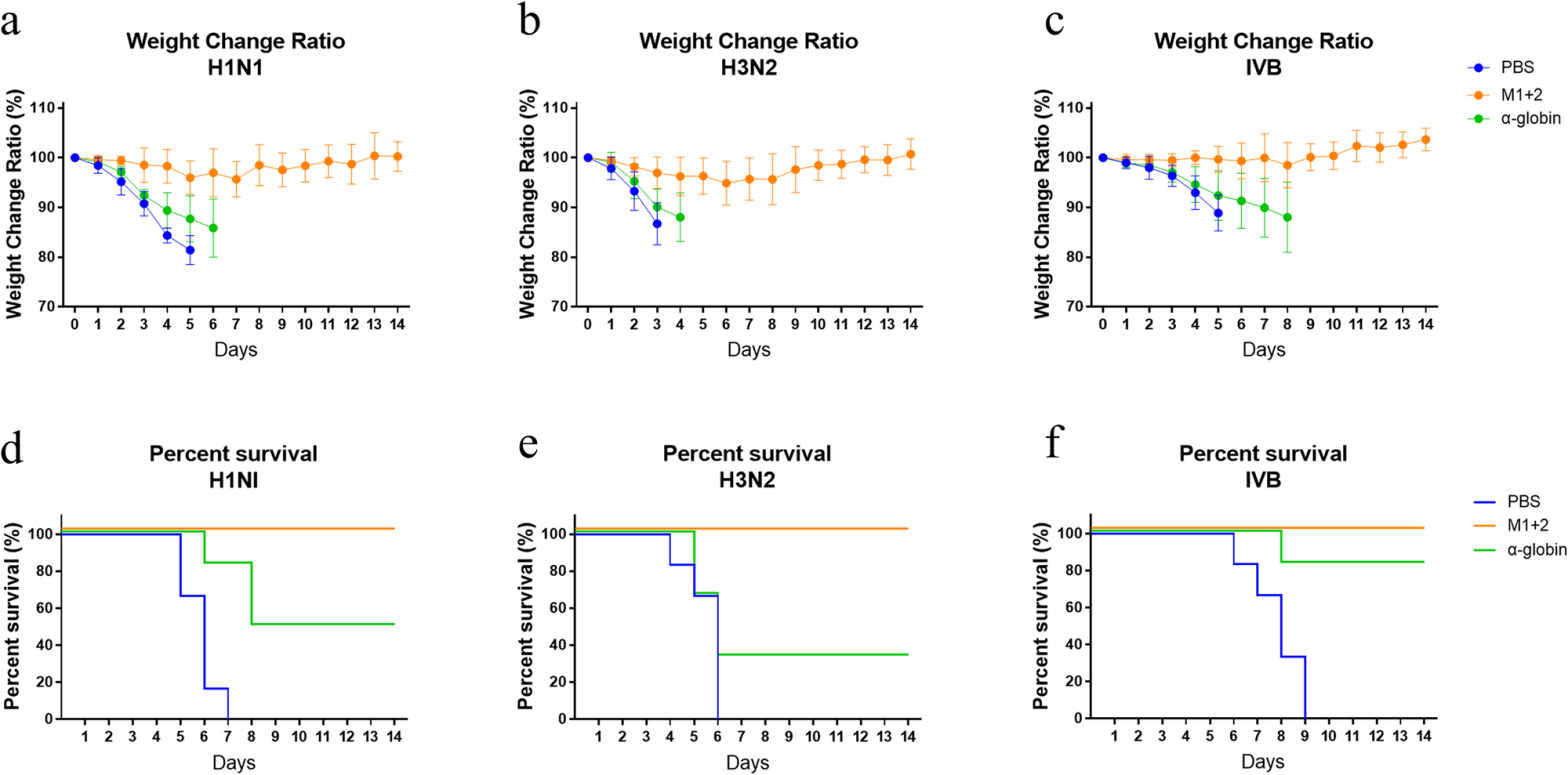
M1 + 2 UTR enables broad protection against seasonal influenza challenges. Mice were immunized twice with 0.1 μg of mRNA-LNPs containing the M1 + 2 or α-globin UTR at a 14-day interval (Day 0 and Day 14), followed by viral challenge on Day 28. Body weight changes after challenge with H1N1 (A/California/07/2009, CA09) (**a**) H3N2 (A/Yangzhou/201/2019) (**b**) or IBV (B/Washington/02/2019) (**c**). Mice were monitored daily for 14 days post-challenge; data are presented as mean percentage of initial body weight. Corresponding survival rates over the 14-day observation period after challenge with H1N1 (**d**) H3N2 (**e**) or IBV (**f**). Data are presented as mean ± SD (*n* = 6).

To further assess the broad applicability of this dose-sparing effect, we generated corresponding HA mRNA-LNPs against H1N1 (CA09), H3N2, or IBV influenza strains incorporating either M1 + 2 or α-globin UTR, then administered the mRNA-LNPs at 0.1 μg per mouse per vaccination, respectively. After challenge with each respective virus, mice immunized with the M1 + 2 UTR-containing vaccine showed no weight loss and achieved complete survival against all three strains (Fig. 7). In contrast, those receiving the α-globin UTR vaccine exhibited significant weight loss and partial mortality across challenge models. These collective findings indicate that the M1 + 2 UTR not only enhances immunogenicity and protective efficacy at low doses but also offers a promising strategy for developing broadly protective, dose-sparing mRNA vaccines against diverse seasonal influenza viruses.

## Discussion

Although mRNA vaccines have demonstrated remarkable success during the COVID-19 pandemic, opportunities for optimization remain, particularly in enhancing translational efficiency and modulating immunogenicity^38^. Current strategies for improving mRNA vaccines include refining lipid nanoparticle (LNP) delivery systems, optimizing coding sequences, and engineering untranslated regions (UTRs) to increase antigen expression and decrease undesirable immuno-stimulation^28,39^. For instance, Sample et al. employed deep learning to analyze over 300,000 human 5’UTRs and identified sequence features that improve ribosome loading and translation efficiency, but in vivo verification is still needed^38^. Other studies emphasize the importance of mRNA secondary structures in regulating stability and immunogenicity, yet the underlying mechanisms of immune sensing remain incompletely elucidated^28,39^. Interestingly, the SARS-CoV-2 mRNA vaccine candidate CV2CoV, which employs chimeric UTRs and unmodified nucleotides, was shown to enhance innate and adaptive immune responses without compromising translation^40^. These findings highlight the potential of UTR engineering to simultaneously enhance mRNA vaccine efficacy and immunogenicity.

The innate immune activity of mRNA vaccines arises largely from the recognition of mRNA molecules by pattern recognition receptors (PRRs), including endosomal Toll-like receptors (TLR3, TLR7, TLR8) and cytosolic sensors such as RIG-I and MDA5^41,42^. This activation triggers type I interferon (IFN-I) signaling and pro-inflammatory cytokine production, which can enhance antigen presentation and promote cellular and humoral immunity^42,43^. However, excessive immuno-stimulation may also contribute to adverse reactions. For example, double-stranded RNA (dsRNA) byproducts produced during in vitro transcription are potent activators of PKR and innate immune pathways, ultimately suppressing translation and increasing reactogenicity^44^. Although nucleoside modifications such as pseudouridine (Ψ) and 1-methylpseudouridine (m1Ψ) can reduce dsRNA formation and mitigate IFN activation, they may also diminish immunostimulatory cues necessary for robust T-cell responses^43,44^. Thus, a central challenge in mRNA vaccine design lies in balancing translational efficiency with appropriate, but not excessive, innate immune activation.

In this study, we hypothesized that modulating mRNA stability through engineered panhandle-forming UTRs could enhance the translational efficiency and immunogenicity of mRNA vaccines. To test this, we screened UTRs derived from the influenza A virus M segment and introduced targeted mutations to strengthen panhandle base-pairing. The resulting lead candidate, M1 + 2, significantly enhanced GFP and luciferase expression in vitro and in vivo compared to unmodified UTRs and the α-globin control. These findings support the utility of structurally engineered viral UTRs in developing more effective and stable mRNA vaccine platforms.

LNPs represent the most clinically advanced delivery platform for mRNA vaccines, as demonstrated by the successful deployment of BNT162b2 (Pfizer/BioNTech) and mRNA-1273 (Moderna) during the COVID-19 pandemic^45^. Typical LNP formulations consist of four key components: phospholipids, cholesterol, polyethylene glycol (PEG)-lipid, and ionizable cationic lipids^46^. These components self-assemble into stable, protective carriers that encapsulate mRNA and facilitate its efficient delivery into host cells^47^. In addition, LNPs can also act as an adjuvant in the agents of vaccines, which inducing innate immune response and thus enhancing the immunogenicity of mRNA vaccines^45^. Upon cellular entry, LNPs enable the release and translation of encoded mRNA into antigenic proteins, which are then presented to the immune system^48^. This process ultimately elicits robust and specific adaptive immune responses, including neutralizing antibody production and T-cell activation, conferring protection against target pathogens^49^.

In this study, hemagglutinin (HA) of influenza A virus (IAV) was employed as the encoded antigen in our mRNA vaccine candidate. HA is a surface glycoprotein essential for viral attachment to host cellular receptors, making it a critical target for neutralizing antibodies and a central component in influenza vaccine development^50,51^. To date, 18 HA subtypes (H1-H18) and 11 neuraminidase (NA, N1-N11) subtypes have been identified, which together define IAV strains such as H1N1 and H3N2^50,52^. Previous studies have established that mRNA-LNPs encoding HA can induce both humoral and cellular immune responses, conferring protection against influenza virus challenge^53,54^. Consistent with these findings, our results demonstrate that HA-based mRNA vaccines elicit robust antibody production and T cell activation. Furthermore, we extend beyond prior work by showing that engineering the mRNA to contain panhandle-forming UTRs, particularly the M1 + 2 variant, significantly enhances translational efficiency and immunogenicity. As shown in Fig. 4d, 4e, vaccination with M1 + 2 UTR-containing mRNA elicited significantly higher hemagglutination inhibition titers compared to the α-globin control, demonstrating that enhanced translation efficiency translates directly into improved humoral immunity. Consistent with this, ELISPOT analysis revealed detectable antigen-specific T cell responses following immunization with M1 + 2 vaccines, further supporting the improved immunogenicity conferred by structural optimization.

Our ELISPOT analysis, conducted 24 hours after the booster immunization, revealed a modest but specific IFN-γ T cell response. The relatively low spot counts are likely attributable to the early time point of analysis, which precedes the peak of clonal T cell expansion typically observed at day 7-10. Nevertheless, the clear difference between antigen-stimulated and mock-stimulated wells, coupled with the statistical significance across groups, confirms the successful induction and rapid recall of antigen-specific cellular immunity. The enhanced early recall response observed with the M1 + 2 UTR underscores its potential in vaccine designs where rapid anamnestic responses are critical for protection.

Studies have indicated that panhandle structures can form a 5’ppp-dsRNA motif recognized by RIG-I, playing a critical role in the recognition of viral genomes during innate immune activation^55^. Further evidence suggested that panhandle structures formed by the complementary UTRs of negative-strand RNA viruses can be directly bound by RIG-I, thereby activating the host innate immune pathways^23,56^. Activation of the RIG-I signaling cascade induces type I interferons (IFN-Ⅰ) and proinflammatory cytokines such as IL-1, IL-6 and IL-10, which are essential for promoting T cell differentiation and enhancing both cellular and humoral immune responses elicited by mRNA vaccines^57,58^.

Consistent with this, our Luminex assay results showed that mRNA vaccines incorporating panhandle-forming UTRs significantly elevated serum levels of multiple cytokines, including IFN-γ, CXCL-10, CCL-2, IL-6, and IL-10, compared to the α-globin control which lacks a panhandle structure. Notably, sequence modifications within the UTR did not compromise this immunostimulatory capacity, and the M1 + 2 variant exhibited a particularly robust immune-activating profile. IFN-γ plays a pivotal role in antiviral defense and immune regulation, functioning as a critical mediator in both innate and adaptive immunity^59^. It potentiates the activation of natural killer (NK) cells, macrophages, T helper 1 (Th1) cells, and cytotoxic T lymphocytes (CTLs), and thus orchestrates subsequent immune defense mechanisms^60,61^. CXCL-10, which is also known as IFN-γ-inducible protein 10 (IP-10) for the secretion in response to IFN-γ stimulation, exerts potent chemotactic effects on monocytes, macrophages, NK cells, dendritic cells (DCs), and T lymphocytes^62–64^. Similarly, CCL-2, which is also referred to as monocyte chemoattractant protein-1 (MCP-1), can specifically mediate monocyte recruitment and subsequent inflammatory processes^65^. Additionally, as classic inflammatory cytokines, IL-6 and IL-10 play essential roles in host defense against viral infections through immunomodulatory functions^66,67^. Collectively, these results indicate that panhandle-forming UTRs can effectively trigger the activation of immune inflammation, thereby enhancing the protective efficacy of mRNA vaccines.

The choice of UTRs in this study was benchmarked against clinically validated elements. The human α-globin UTR, used in our research as a reference, is a key component of authorized mRNA vaccines, serving as the 5’UTR in the Pfizer/BioNTech BNT162b2 vaccine and contributing to the 3’UTR of the Moderna mRNA-1273 vaccine^68–70^. Our data demonstrate that the novel M1 + 2 UTR, engineered from the influenza A virus M segment, drives superior antigen expression and immunogenicity compared to this established gold standard. While a direct head-to-head comparison with the exact, full-length UTR sequences used in these commercial platforms (which often involve proprietary combinations and specific codon optimization) was beyond the scope of this mechanistic study, our results clearly show that the M1 + 2 UTR enhances mRNA performance beyond a key regulatory element shared by leading vaccines. This finding underscores the significant potential of rational UTR design to further improve the efficacy of next-generation mRNA vaccines.

As we have described above, optimizing mRNA stability and translation efficiency offers significant advantages, both in reducing economic costs and in improving safety profile. Through in vitro transfection of EGFP in HEK293T cells and in vivo imaging of Luc-mRNA-LNPs, we demonstrated that incorporating UTRs capable of forming a panhandle structure significantly enhances translation efficiency. To further assess whether this improvement in mRNA translation could elicit a sufficient immune response at minimal vaccine doses, we immunized mice with very low doses of HA-mRNA-LNP. As measured by hemagglutination inhibition assay, HA mRNA carrying the M1 + 2 UTR consistently induced higher levels of HA-specific antibodies compared to the α-globin UTR. The M1 + 2 construct required only a dose of 0.1 μg to produce antibody titers comparable to those achieved by 0.5 μg of the α-globin-based vaccine. Similarly, when evaluating weight change ratios and survival rates following viral challenge, vaccination with HA-mRNA-LNP containing the M1 + 2 UTR provided sufficient immuno-protection at the 0.1 μg dose. In contrast, the α-globin UTR, which does not form a panhandle-like structure, failed to confer effective protection at the same dosage. These findings demonstrate that enhancing translation efficiency through the introduction of the M1 + 2 mutated UTR to form a panhandle-like secondary structure can effectively optimize the dosing regimen of mRNA vaccines. The resulting dose-sparing effect offers multiple significant advantages, including lower production costs that enhance vaccine affordability, improved safety profile with reduced reactogenicity, expanded manufacturing capacity enabling more rapid global distribution, potential for streamlined development of multivalent vaccine formulations, and increased flexibility in addressing emerging viral variants through adaptable vaccination strategies.

In conclusion, our investigation demonstrated that engineered panhandle-forming UTRs significantly enhance the translational efficiency of mRNA vaccines. The M1 + 2 UTR variant not only promoted robust antigen expression in vitro and in vivo but also elicited potent innate and adaptive immune responses conferring protection against multiple seasonal influenza strains even at ultralow doses. These findings underscored the potential of rational UTR design to simultaneously increase vaccine efficacy and achieve substantial dose reduction. By improving translational performance and enabling dose-sparing effects, this strategy may help lower production costs and enhance the accessibility of mRNA vaccine platforms. Future studies should explore the applicability of structured UTRs in other mRNA-based vaccines, with the ultimate goal of facilitating broader clinical deployment against diverse infectious diseases. Additionally, systematic evaluation of delivery parameters, such as injection volume, could provide further insights for optimizing dose-sparing vaccination strategies.

## Methods

### Viruses, cells, and animals

The H1N1 influenza A virus strains A/Puerto Rico/8/1934 (H1N1-PR8), H1N1 influenza A virus strains A/California/07/2009 (H1N1-CA09), H3N2 influenza A virus strains (A/Yangzhou/201/2019) (H3N2), and influenza B virus strains B/Washington/02/2019 (IBV) were used in this study. The A/Yangzhou/201/2019 (H3N2) strain was kindly provided by Professor Pinghu Zhang (Yangzhou University) and has been previously demonstrated to be pathogenic in mice^71^. The B/Washington/02/2019 (IBV) strain was kindly provided by Professor Yuelong Shu (Shenzhen Campus of Sun Yat-sen University). Influenza viruses were propagated in specific pathogen-free (SPF) embryonated chicken eggs and the allantoic fluid were collected and stored at -80 °C until use. Viral titres were determined using standard plaque assay.

Human embryonic kidney 293 T (HEK293T) cells were cultured in DMEM high-sucrose medium (Hyclone, USA) supplemented with 10% fetal bovine serum (FBS; VivaCell, China) and 1% penicillin/streptomycin (Solarbio, China). Madin-Darby canine kidney (MDCK) cells were cultured in MEM medium (Hyclone, USA) supplemented with 10% FBS (VivaCell, China) and 1% penicillin/streptomycin (Solarbio, China).

6–8 weeks old female SPF BALB/c mice were purchased from Huachuang Sino Medical Technology Co., Ltd (Jiangsu, China, SCXZ (Su)2020-0009). All experimental procedures were approved by the Institutional Animal Care and Use Committee at Nanjing University of Chinese Medicine (2024-LY-kt060) (Approved on February 21, 2024). All animal experimental tests were carried out in accordance with the 2016 standards of laboratory animal in China and other related regulations in Animal Welfare Act.

### mRNA production

The EGFP, Renilla luciferase, and HA genes from H1N1-PR8, H1N1-CA09, H3N2, or IBV, with flanked 5’ and 3’ UTR, were cloned into pVAX1 vector as PCR and in vitro transcript plate. A panel of 5’ and 3’ UTR forming a panhandle structure from different virus were screened. The 5’ and 3’ UTR sequence used in this research was derived from the M gene fragment of influenza A virus and denoted as M. To enhance the stability and translation of mRNA, an array of mutations at 3’ UTR were made to increase the number of base-pair, and named them as M1, M2, M3, M4 or M1 + 2.

T7 High Yield RNA Transcription Kit (Novoprotein, China) was used to perform the in vitro transcription according to the manufacturer’s instructions by using linearized DNA as templates. In this research, 1-methylpseudouridine-5’-triphosphate (Novoprotein, China) was used instead of uridine triphosphate for mRNA synthesis. The synthetic mRNA was subsequently capped using the Cap 1 Capping System (Novoprotein, China) and polyadenylated with the E. coli Poly(A) Polymerase tailing system (Vazyme, China). The mRNA was then purified using LiCl (Thermo, USA). The concentration and purity of the capped and tailed mRNA were determined by measuring absorbance at 260 nm with a Nanodrop 8000 spectrophotometer (Thermo, USA).

### mRNA-LNP preparation

SM102 (Macklin, China), DSPC (Merck, Germany), DMG-PEG2000 (Merck, Germany), and cholesterol (Macklin, China) were separately dissolved in anhydrous ethanol to a concentration of 16 mM, and were then mixed according to the volumetric ratio of SM102: DSPC: DMG-PEG2000: cholesterol as 50: 10: 1.5: 38.5 to prepare Lipid nanoparticle (LNP). Subsequently, the mRNA diluted in citric acid buffer (pH=4.0) was mixed with the LNP through microfluidic nano-preparator (INano L+, Micro&Nano) at a flow rate ratio of 3:1. The N:P ratio was set to 6:1, and the total flow rate was set at 16 mL/min. The mRNA-LNP solution was diluted with PBS upon packaging and was then ultra-filtered to purify using a 100 kD ultrafiltration tube (Sigma-Aldrich, Germany). Following purification, the Quant-iT RiboGreen RNA Reagent Kit (Thermo, USA) was used to assess the encapsulation rate and concentration of mRNA-LNP nanoparticles. The particle size and homogeneity were determined using a zeta potential analysis system (Zetasizer pro, Malvern Panalytical). To detect the surface morphology of prepared mRNA-LNP, samples were negatively stained with 2% uranyl acetate and observed under transmission electron microscopy (TEM; HT7800, Hitachi).

### Cell transfection with EGFP mRNA

HEK293T cells were seeded at a density of 1 × 10^5^ cells/mL and cultured overnight at 37 °C in a 5% CO_2_ incubator. Prior to transfection, the DMEM complete medium was replaced with Opti-MEM medium (Gibco, USA), and the cells were incubated in the CO_2_ incubator at 37 °C for 30 min. Then, the prepared mRNA-LNP nanoparticles were added into cell culture, and the cells were incubated for an additional 6 h at 37 °C. Subsequently, equal volume of DMEM complete medium was added into the cell culture plates. After the cells were incubated in the CO_2_ incubator at 37 °C for 24 h, cells were visualized using an Observe7 fluorescence microscope (Observer 7, Zeiss), and the fluorescence was quantitatively analyzed using ImageJ software (V1.8.0.112).

### In vivo bioluminescence imaging

6–8 weeks old female SPF BALB/c mice were divided into five groups with three mice in each group. Luc-mRNA-LNP containing α-globin, M, M1, M2, or M1 + 2 UTRs were respectively intramuscular-injected into the BALB/c mice at a fixed volume of 0.1 mL (containing 0.2 μg mRNA). For intramuscular administration, mice received a single injection of mRNA-LNP into the hindlimb muscles, which is the standard and most commonly described site for intramuscular injections in murine models^72^. After 48 hours post injection, mice were injected intraperitoneally with 0.2 mL of luciferase substrate (Promega, USA). The in vivo imaging was conducted at 10 min after intraperitoneal injection, then the bioluminescence signal was subsequently quantified using imaging system (X5-SE, Tanon) and analyzed using GraphPad Prism 7.0.

### Standard plaque assay for influenza virus

The plaque assay was performed as described previously^73,74^. MDCK cells were seeded into 6-well plates at a density of 6 × 10⁵ cells/well and incubated at 37 °C with 5% CO₂ until a confluent monolayer formed. The influenza virus stock was serially diluted in 10-fold increments using serum-free DMEM. After removing the culture medium (MesGen, China) and washing with PBS, 500 μL of diluted virus suspensions were added to designated wells. The plates were incubated for 1 h at 37 °C with gentle agitation every 15 min to ensure uniform viral adsorption. After viral adsorption, the inoculum was aspirated, and an agarose overlay containing TPCK-treated trypsin was applied. The overlay was prepared by mixing equal volumes of 2× virus maintenance medium (containing 2× DMEM (Gibco, USA), BSA (Absin, China), NEAA (Gibco, USA), and TPCK-trypsin (Sigma-Aldrich, Germany)) and 1.6% agarose solution (Solarbio, China), resulting in a 0.8% agarose overlay with 1× maintenance medium. The pre-warmed overlay mixture was gently added onto the cells with 2 mL/well. The overlay was allowed to solidify at room temperature, and inverted incubating for 72 h. Cells were fixed with formalin (Biosharp, China) and stained with 0.1% crystal violet (Biosharp, China). The plaque-forming units (PFU/mL) were calculated using the formula: PFU/mL = (Mean plaque count per well)/(Virus inoculum volume [mL] × Dilution factor).

### mRNA vaccination and challenge with influenza virus

6–8-week BALB/c mice were randomly allocated into experimental groups. Each vaccination group received an intramuscular injection of 0.1 mL of the corresponding mRNA-LNP candidate vaccine, while the control group was administered an equivalent volume of PBS. All mice were immunized twice at a 14-day interval. Fourteen days post the boost immunization, mice were anesthetized by inhalation of isoflurane and then intranasally challenged with 50 μL of PBS containing 10 mouse-lethal doses of 50% (MLD₅₀) influenza virus. Body weight and survival were monitored daily for 14 days post-challenge. Any mouse that lost more than 25% of its initial body weight was humanely euthanized by cervical dislocation to prevent unnecessary suffering, and this was recorded as a mortality endpoint.

### Hemagglutination inhibition assay

Serum samples were subjected to a two-fold serial dilution in V-bottom 96-well plates (Biosharp, China) using PBS as the diluent. After dilution, 25 μL of diluted serum was dropped into each well. Subsequently, 4 hemagglutination units (4HAU) of viruses in 25 μL PBS were added to each well. Plates were gently tapped to mix and incubated at room temperature for 30 min. Following incubation, 50 μL of 1% chicken erythrocyte suspension (Sbjbio, China) was added to each well. Plates were mixed again by gentle agitation and kept at room temperature for 40 min. Hemagglutination inhibition was assessed visually by observing the formation of a distinct erythrocyte pellet or diffuse lattice. The HI titer was defined as the reciprocal of the highest serum dilution that completely inhibited hemagglutination.

### Flow cytometry analysis

For analysis of immune cells, a separate cohort of mice was humanely euthanized by cervical dislocation 24 h after the second immunization. Spleens were then aseptically harvested for immediate processing into single-cell suspensions for flow cytometry analysis. Single-cell suspensions of splenic lymphocytes were isolated using a commercial lymphocyte isolation solution (Dakewe, China). To minimize nonspecific binding, cells were pre-incubated with anti-mouse CD16/CD32 monoclonal antibody (Cat No. 553141, BD Biosciences, USA) for 15 min at 4 °C to block Fc receptors. Subsequently, cells were stained with fluorochrome-conjugated antibodies, PE-anti-mouse CD3e (Cat No. 553063, BD Biosciences, USA), APC-anti-mouse CD4 (Cat No. 553051, BD Biosciences, USA), and FITC-anti-mouse CD8a (Cat No. 553030, BD Biosciences, USA), for 30 min on ice in the dark. Following staining, cells were washed twice with ice-cold FACS buffer (PBS supplemented with 1% bovine serum albumin) and resuspended in 300 μL of the same buffer for analysis. Flow cytometry was performed using a BD FACSCanto™ II system (BD Biosciences, USA), and a minimum of 10,000 live lymphocyte-gated events per sample were acquired. Data analysis was conducted using FlowJo software (V10.6.2) with appropriate compensation controls and gating strategies based on unstained and single-color controls.

### Enzyme-LInked Immunospot (ELISPOT) assay

The ELISPOT assay was performed to quantify antigen-specific IFN-γ-secreting splenocytes. Pre-coated anti-mouse IFN-γ ELISPOT plates (Dakewe, China) were pre-treated with RPMI-1640 medium (Hyclone, USA) containing 10% FBS for 10 min at room temperature. Splenocytes were seeded into the plates at a density of 1 × 10^5^ cells/well and stimulated with either 0.1 μg/mL PepTivator Influenza A (H1N1) HA peptide pool (Miltenyi Biotec, Germany) to assess antigen-specific responses, 5 μg/mL concanavalin A (ConA; Sigma-Aldrich, Germany) as a positive control, or RPMI-1640 medium alone (negative control). Plates were incubated overnight at 37 °C, 5% CO_2_ incubator. Following incubation, cells were removed, and plates were washed six times with washing buffer. Biotinylated detection antibody and streptavidin-horseradish peroxidase (HRP) conjugate were sequentially added according to the manufacturer’s protocol. Spots were developed using 3-amino-9-ethylcarbazole (AEC) substrate and quantified with an AID ELISPOT Reader System (Classic ELR80, AID GmbH).

### Luminex

The levels of inflammatory cytokines/chemokines in serum were quantified using a commercially available Luminex multiplex assay kit (R&D Systems, USA) according to the manufacturer’s instruction. The panel included ten inflammation-associated biomarkers, interferon-γ (IFN-γ), C-X-C motif chemokine ligand 10 (CXCL10), C-C motif chemokine ligand 2 (CCL2), interleukin-1β (IL-1β), IL-2, IL-4, IL-6, IL-10, tumor necrosis factor-α (TNF-α), and granulocyte-macrophage colony-stimulating factor (GM-CSF). Of the ten cytokines analyzed, six (IFN-γ, CXCL10, CCL2, IL-6, TNF-α, and GM-CSF) demonstrated measurable levels above the assay’s lower limit of detection, and the data from these six cytokines were visualized as a heatmap.

### Statistical analysis

All statistical analyses were performed using GraphPad Prism 7. Data are presented as mean ± SD, with the number of mice per group specified in the corresponding figure legends. For comparisons between two groups, an unpaired two-tailed Student’s *t* test was applied. One-way ANOVA was conducted, followed by Dunnett’s post hoc test to evaluate differences between experimental groups and the control group. *P*-value < 0.05 was considered statistically significant.

## Supplementary information

Supplementary information

## Data Availability

All data supporting the findings of this study are included in this manuscript or its supplementary information files.
