# Supplementary material for: Influenza mRNA vaccine with engineered panhandle-forming UTRs provides potent, dose-sparing protection against seasonal influenza viruses
Source: NPJ Vaccines. 2026 Apr 27;11:132. doi: 10.1038/s41541-026-01463-3 (PMC13323722; doi:10.1038/s41541-026-01463-3)
Supplement: Supplementary file 1 — Supplementary information [file 41541_2026_1463_MOESM1_ESM.pdf]

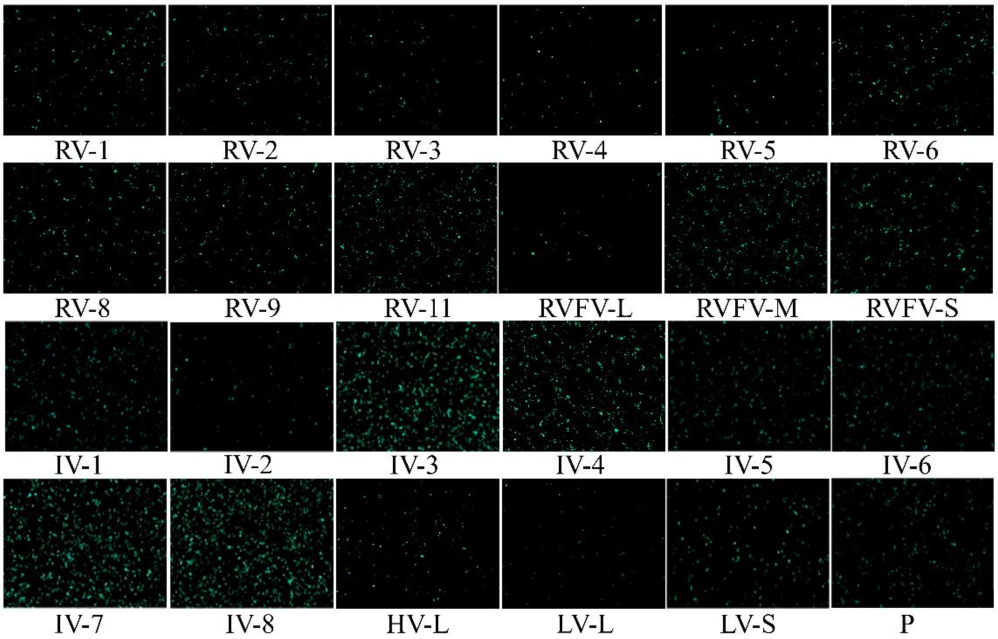

Fig. S1 | Initial EGFP-based screening of viral UTR variants.

Representative fluorescence images of HEK-293T cells taken at 24 h after transfection with EGFP-encoding mRNAs containing 23 different viral UTR pairs. The EGFP mRNA without any UTR sequences was transfected as a positive control.

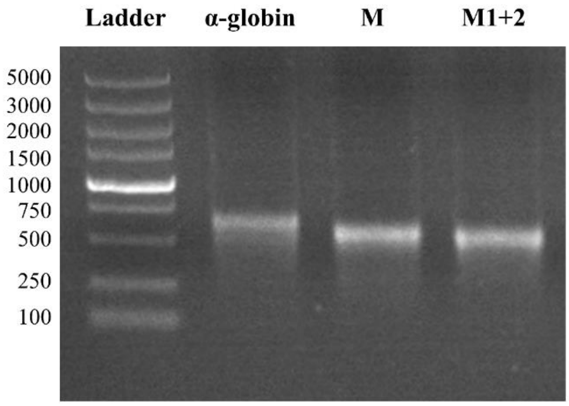

Fig. S2 | Integrity analysis of mRNA with different UTRs.

Representative agarose gel electrophoresis image of EGFP-encoding mRNAs containing α-globin, M, or M1+2 UTRs. mRNAs were synthesized by in vitro transcription, purified, purified, and loaded to individual lane as indicated. The gel was stained with nucleic acid dye and visualized under UV light. All mRNAs show a single, clear band with no visible degradation smears, indicating comparable integrity across UTR variants.

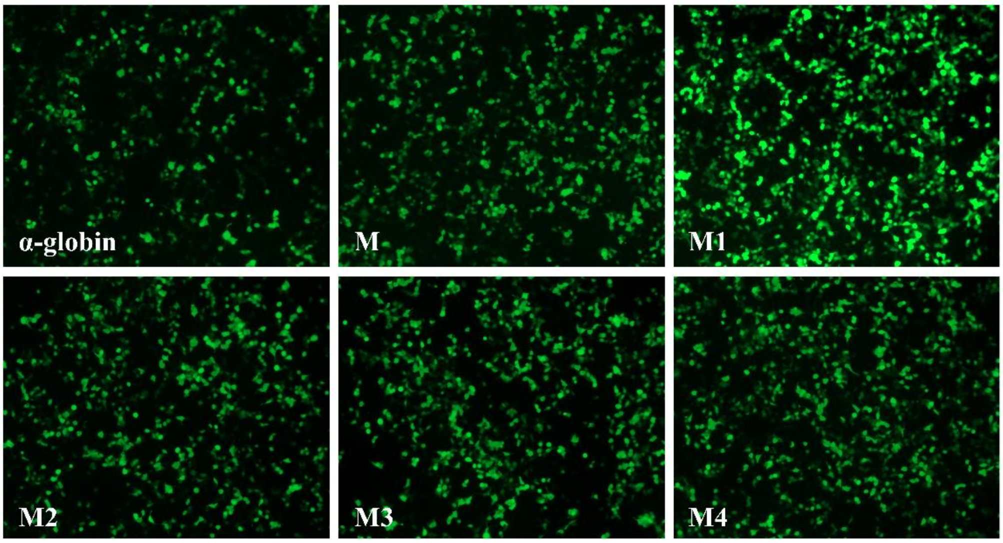

Fig. S3 | EGFP-based screening of M1, M2, M3, and M4 single-point mutants derived from influenza M segment UTR.

Representative fluorescence images of HEK-293T cells taken at 24 h after transfection with EGFP-encoding mRNAs containing the prototype M segment UTR (M), single mutants (M1, M2, M3, and M4), or the α-globin control UTR. Cells were transfected with 2 μg of mRNA-LNPs per well.

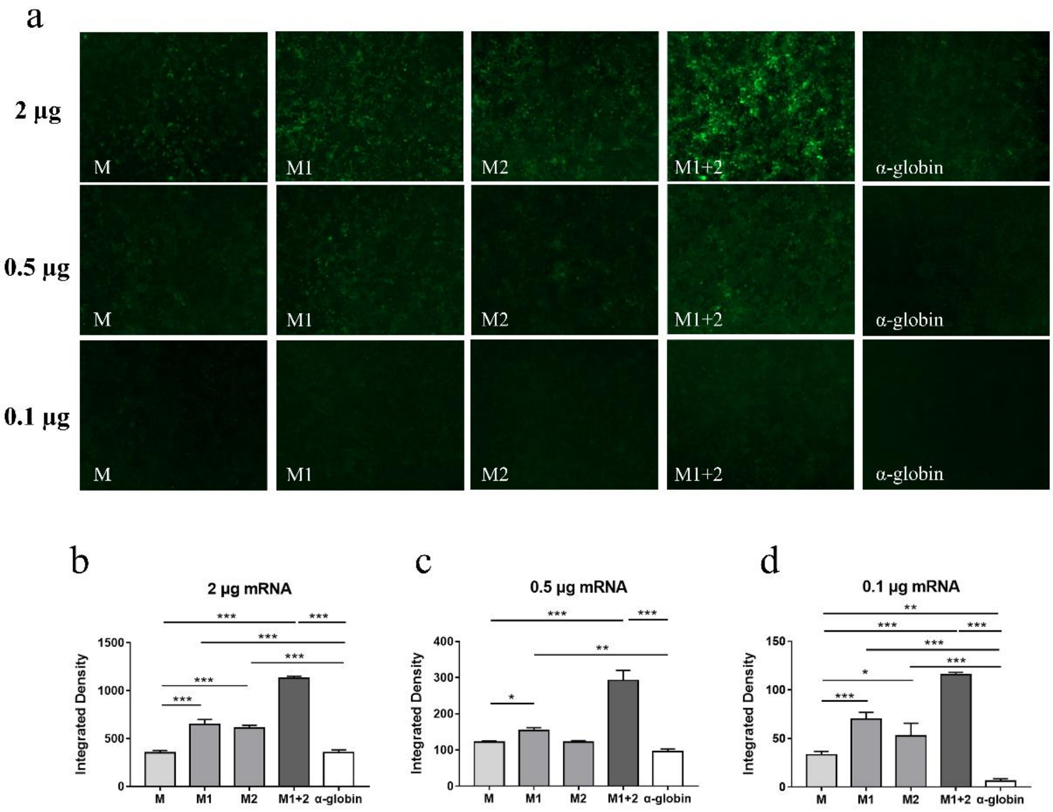

Fig. S4 │ Validation of M1+2 UTR-enhanced EGFP expression at multiple doses.

(a) Representative fluorescence images of HEK-293T cells taken at 24 h after transfection with 2, 0.5, or 0.1 μg of EGFP-encoding mRNA-LNPs containing the prototype M segment UTR (M), single mutants (M1, M2), the M1+2 variant, or the α-globin control UTR (related to Fig. 2c). (b–d) Quantification of total fluorescence intensity (integrated density per image field) measured by ImageJ for cells transfected with 2 μg (b), 0.5 μg (c), or 0.1 μg (d) mRNA. Data are presented as mean ± SD (n = 3). *: $P<0.05$, **: $P<0.01$, ***: $P<0.001$.

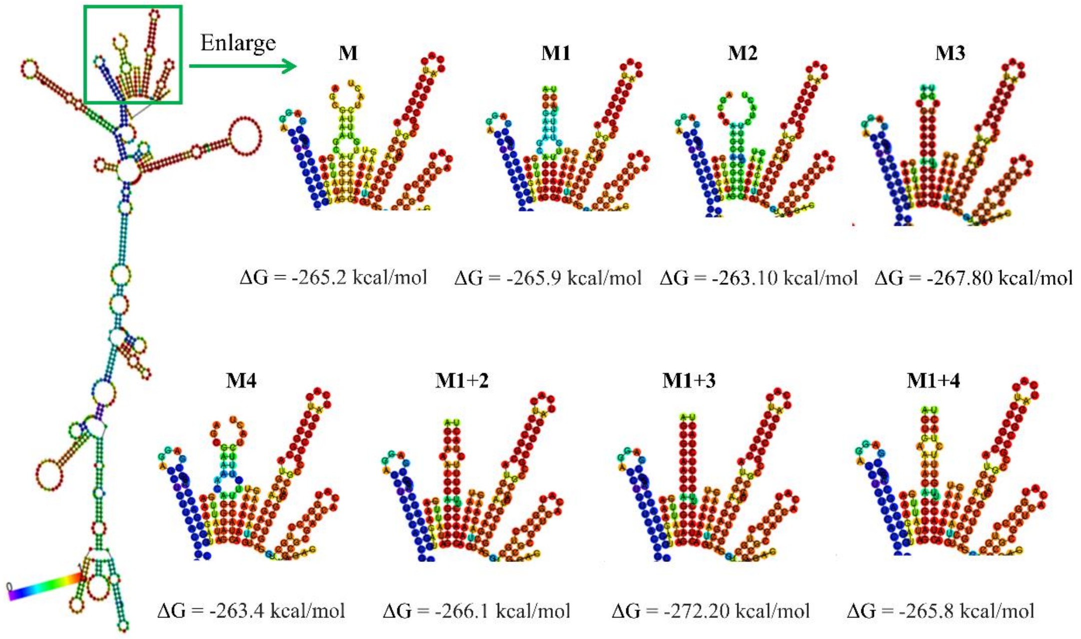

Fig. S5 | Computational calculation of secondary structures and thermodynamic parameters for M segment-derived UTR variants.

Predicted secondary structures and minimum free energy (ΔG) values for M segment-derived UTR variants used in this study. Structures were calculated using the RNAfold WebServer with default parameters. The predicted ΔG value (kcal/mol) is indicated below each structure. Variants shown include the prototype M segment UTR (M), single mutants (M1, M2, M3, M4), and combination mutants (M1+2, M1+3, M1+4).

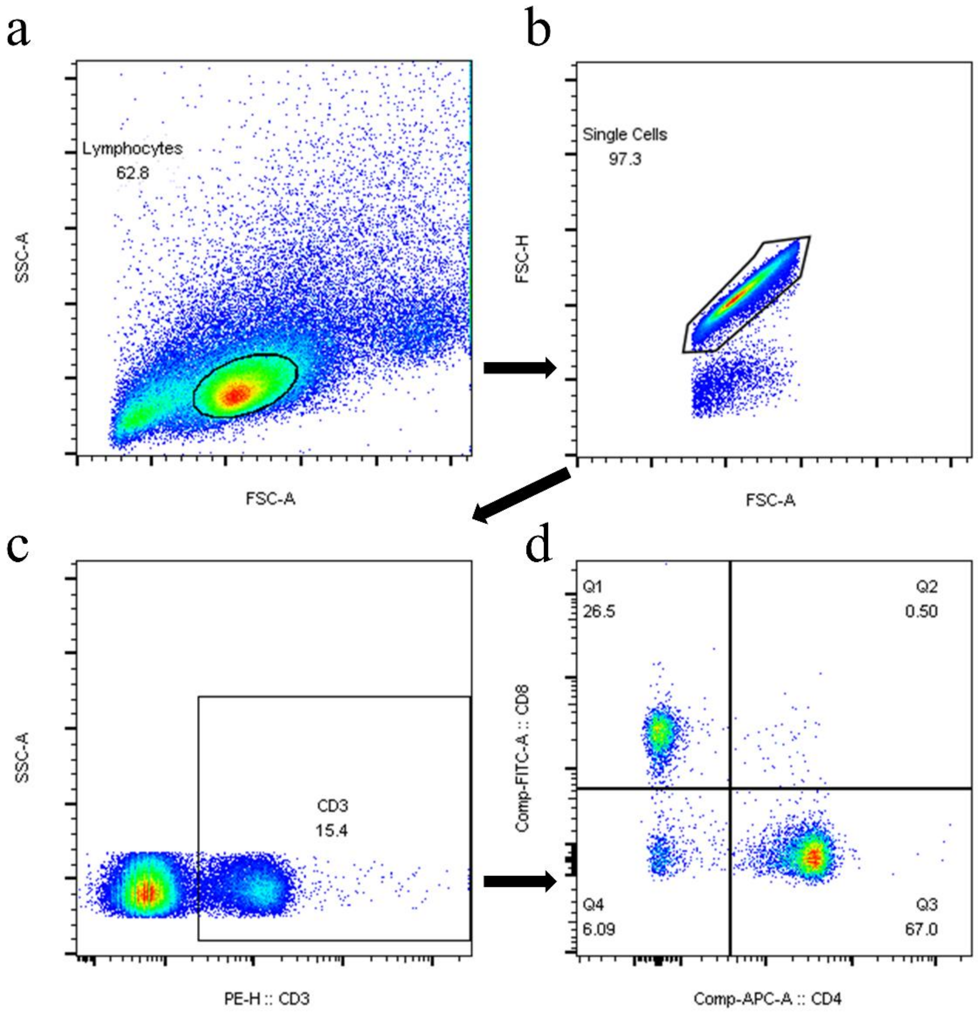

Fig. S6 | Gating strategy for flow cytometric analysis of T cell subsets.

Representative sequential gating strategy used to identify $CD3^+$, $CD4^+$, and $CD8^+$ T cell subsets from splenocytes (related to Fig. 4c). Data were analyzed using FlowJo software.

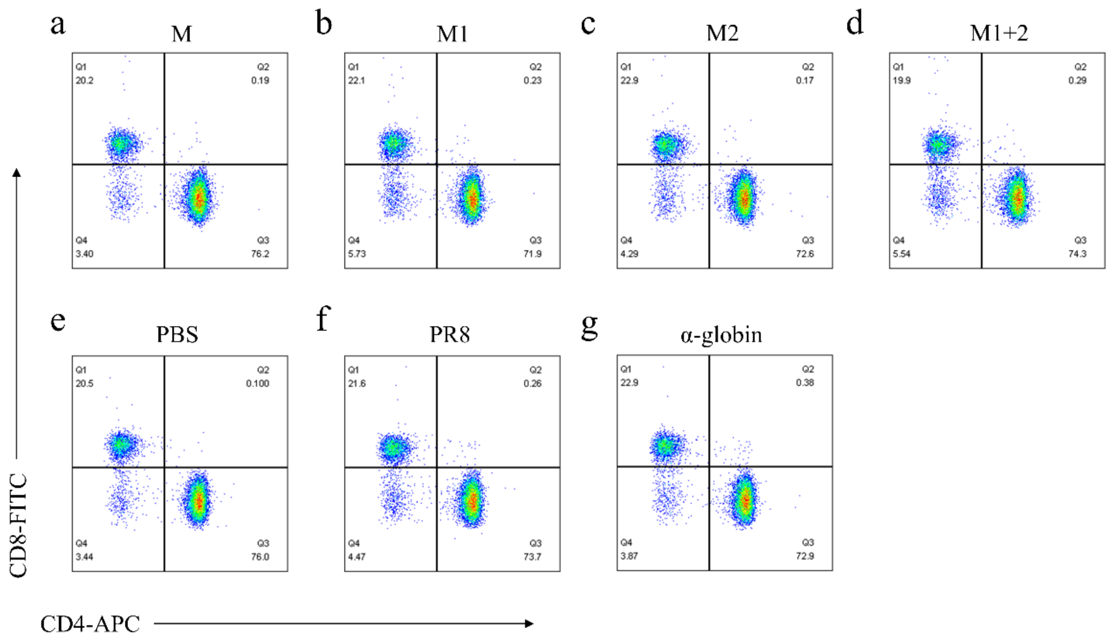

Fig. S7 | Flow cytometric analysis of CD4$^+$ and CD8$^+$ T cell subsets.

Representative flow cytometry plots showing CD4$^+$ and CD8$^+$ T cell populations in splenocytes isolated from mice (related to Fig. 4c). Cells were gated on CD3$^+$ lymphocytes as shown in Fig. S6, and the percentages of CD4$^+$ and CD8$^+$ subsets within the CD3$^+$ population are displayed. Data were analyzed using FlowJo software.

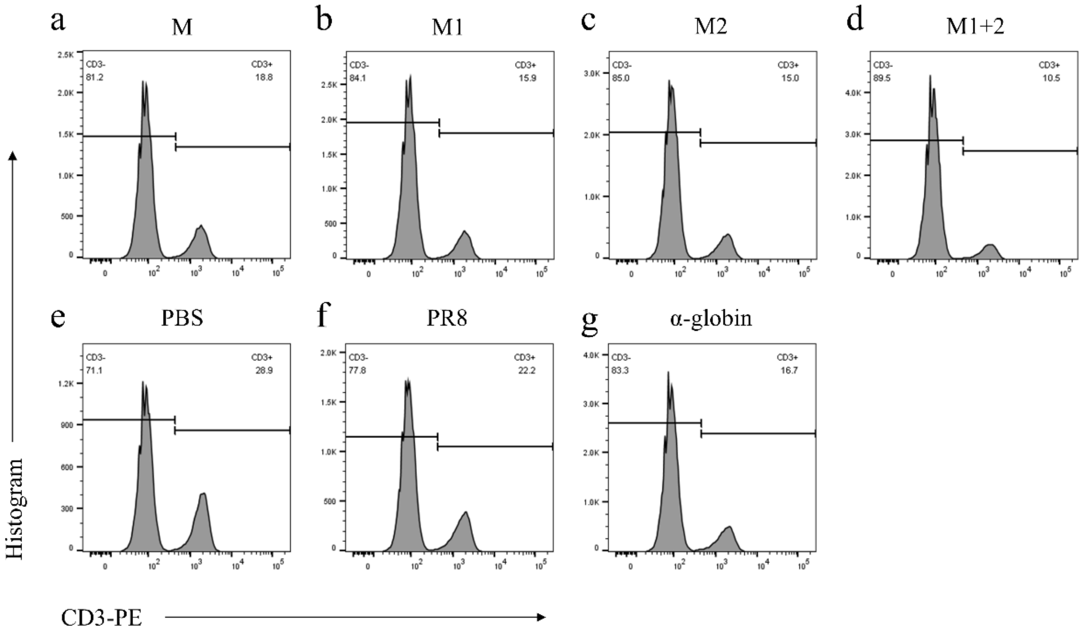

Fig. S8│Representative histograms illustrating CD3⁺ T cell gating and population distributions.

Representative flow cytometry histograms showing CD3⁺ T cell proportions in splenocytes isolated from mice (related to Fig. 4c). Cells were gated on lymphocytes as shown in Fig. S6, and CD3⁺ frequencies are displayed for all experimental groups. Quantitative analysis of CD3⁺ percentages across groups is presented in Fig. 4c; representative histograms are shown here to illustrate the gating and population distributions. Data were analyzed using FlowJo software.

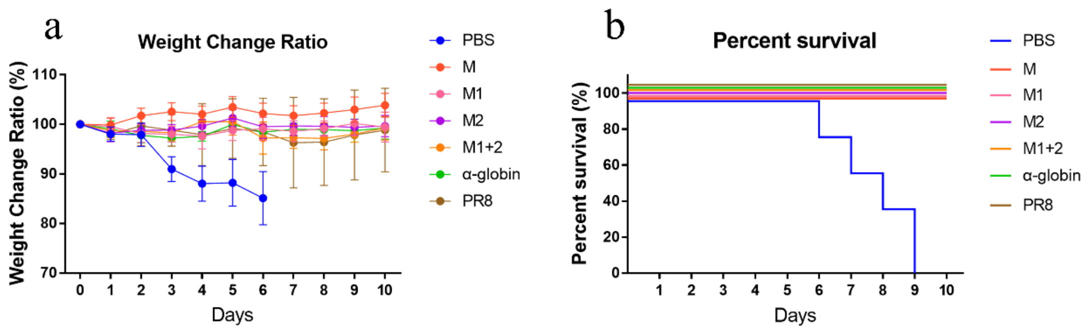

Fig. S9 | Immune protection of mRNA-LNPs against PR8 challenge at standard dose.

(a) Body weight changes of mice (n = 5 per group) immunized with 2 μg of mRNA-LNPs containing the indicated UTR variants. Mice were challenged intranasally with PR8 (H1N1) virus at Day 28 post-prime immunization, and body weight was monitored daily for 10 days thereafter. Data are presented as mean percentage of initial body weight ± SD. (b) Survival rates of the same mice over the 10-day observation period following viral challenge.

Table S1. Complete sequences of 5'- and 3'-UTRs used in the initial UTR screening.

	5'UTR	3'UTR
RV-1	5'-GGCTTTTAAAGCGTCTCA	5'-ACTCTGCGAAATTTTCGG
RV-2	5'-GGCTATTAAAGGCTCA	5'-GGTCATATCTCCACAGTGGGGTTGGCGT
RV-3	5'-GGCTTTTAAAGCAGTACTAGTAGTGCGTTTTACCTCTGATGGTGTAAGC	5'-GGTCACATCATGACTAGTGTGTTAAGTTTCTAGC
RV-4	5'-GGCTATAAA	5'-GGTCACATCCTCAATAGCGTTCTCACAAT
RV-5	5'-GGCTTTTTTTATGAAAAGTCTTGTGGAAGCC	5'-GGTTCACATTCTATGCTGCCTAGGCGCTACTCTAGTACAGGGAGTCTTGTAGTGGCAAATGTGACCTTTCTTTTAC
RV-6	5'-GGCTTTAAAACGAAGTCTTCGAC	5'-GGTCACATCCTCTCACTACATCATTCAGACAGTTACTCTACGTAGCGAACATGAAATCATGTCCTTACTTGAAGAGTCTGAATGACTTGATGTAGCTACATGCTAACTAAGATTGGATACCAGATGTTTAGTCTGGTCC
RV-8	5'-GGCTTTTAAAGCGTCTCAGTCGCCGTTTGAGCCTTGCGGTGTAGCC	5'-GGTCACATAAGCGCTTTCTATTCTTGCTTCGTCATCATCATTCTCGAATCGAAAGTGAA
RV-9	5'-GGCTTTAAAAGAGAGAATTTCCGTCTGGCTAACGGTTAGCTCCTTTTA	5'-GGTCACATCGAACAATTCTAATCTAAGATAT
RV-11	5'-GGCTTTAAAAGCGCTACAGTG	5'-GGTCACAAAACGGGAGTGGGGAGCTCCCTAGTGCGTCCTCAGGTCG
RVFV-L	5'-ACUAUGAUCGUAUCACGAUGU	5'-CUGGUCAUUCGUUUCAGUCCGAA

RVFV-M	5'-AUUAAACCCCACCACCCCACUCCGUCGUCGUC	5'-CCCACCACCCCACUCC
RVFV-S	5'-AUCCCCCACCCCCCAC	5'-CCCAGGGGGUUGGGGG
IV-1	5'-AGCGAAAGCAGGTCAATTATATTCAAT	5'-AGTAGAAACAAGGTCGTTTTTAAACTATTCGACA
IV-2	5'-AGCGAAAGCAGGCAAACCATTTGA	5'-AGTAGAAACAAGGCATTTTTTCATGAAGGACAAGCTAAATTCA
IV-3	5'-AGCGAAAGCAGGTACTGATCCAAA	5'-AGTAGAAACAAGGTACTTTTTTGGACAGTATGGATAGCAAATAGTAGCACTGCCACAA
IV-4	5'-AGCAAAAGCAGGGGAAAATAAAAACAACCAAA	5'-AGTAGAAACAAGGGTGTTTTTCCTCATATCTCTGAAATTCTAATC
IV-5	5'-AGCAAAAGCAGGGTAGATAATCACTCACTGAGTGACATCAAAATC	5'-AGTAGAAACAAGGGTATTTTTCT
IV-6	5'-AGCGAAAGCAGGGGTTTAAA	5'-TCTGTTCAAAAAACTCCTTGTTTCTACT
IV-7	5'-AGCGAAAGCAGGTAGATATTGAAAG	5'-AAAACTACCTTGTTTCTACT
IV-8	5'-AGCAAAAGCAGGGTGACAAAGACATA	5'-AGTAGAAACAAGGGTGTTTTTTATTATTAAATAAGCTGAAACGAGAAAGTTC
HV-L	5'-TAGTAGTAGGACTTCCGAGATAGAGAAAGATCAATCAAAAAAA	5'-TAGTAGTATGCTCCGGAAAAAGAGCACTGC
LV-L	5'-CGCACCGGGGATCCTAGGCAATTTTGTGGATCTTTGCTGATACTTCATTCTGCACTCAAAGCCGCATCCGGCAAGAGTC	5'-CGCACCGGGGATCCTAGGCATTGTGACTCTCTCTTCTGAGGAACAAGTGTGTGG

LV-S	5'-GCGCACCGGGGATCCTAGGCATTTTTGGTTGCGCAATTCAAGTGTCCTATTTAAA	5'-CGCACAGTGGATCCTAGGCTATTGGATTGCGCTTTGCTTTTGTCATTTTGGCAGATAGTCTCAGTTCTTTGTTGTGTGCATACAACACAACAATCTGG

Table S2. UTR sequences of M segment-derived variants used in core experiments.

	5'UTR	3'UTR
M1	5'-AGTGAAAGCAGGTAGATATTGAAAG	5'-AAAACTACCTTGTTTCTACT
M2	5'-AGCAAAAGCAGGTAGATATTGAAAG	5'-AAAACTACCTTGTTTCTACT
M3	5'-AGCGGAAGCAGGTAGATATTGAAAG	5'-AAAACTACCTTGTTTCTACT
M4	5'-AGCGAAAACAGGTAGATATTGAAAG	5'-AAAACTACCTTGTTTCTACT
M1+2	5'-AGTAAAAGCAGGTAGATATTGAAAG	5'-AAAACTACCTTGTTTCTACT
M1+3	5'-AGTGGAAGCAGGTAGATATTGAAAG	5'-AAAACTACCTTGTTTCTACT
M1+4	5'-AGTGAAAACAGGTAGATATTGAAAG	5'-AAAACTACCTTGTTTCTACT
